# A flexible short protocol in women with poor ovarian response over 40 years old

**DOI:** 10.1186/s13048-020-00761-1

**Published:** 2021-01-05

**Authors:** Xinyue Zhang, Ting Feng, Jihong Yang, Yingying Hao, Suying Li, Yan Zhang, Yun Qian

**Affiliations:** Reproductive Medical Center of the Second Affiliated Hospital of Nanjing Medical University, 121 Jiangjiayuan, 210011 Nanjing, China

**Keywords:** Assisted reproductive technology, poor ovarian response, flexible, ovarian stimulation protocol

## Abstract

**Background:**

Ovarian responsiveness to controlled ovarian stimulation is essential for a successful clinical outcome in assisted reproductive technology (ART) cycles. We aimed to find a suitable new ovulation stimulation protocol for poor ovarian response (POR) patients over 40 years old.

**Methods:**

A retrospective analysis of 488 ART cycles was evaluated from January 2015 to June 2019. Comparisons were made between the flexible short protocol (FSP), routine short protocol and mild stimulation protocol.

**Results:**

Compared with the routine short protocol, the FSP delayed the gonadotropin start time and reduced the total gonadotropin dose per stimulation cycle. At the same time, compared with the mild stimulation protocol, the FSP improved oocyte quality and embryo quality and improved embryo implantation potential after transfer. Furthermore, the use of the FSP reduced the probability of premature ovulation, as it inhibited the premature luteinizing hormone (LH) surge to a certain extent.

**Conclusions:**

The FSP yielded better outcomes than other protocols for patients with POR over 40 years old in our study. However, further prospective studies are needed to provide more substantial evidence and to determine whether the FSP can be successful for both patients over 40 years group and younger POR patients.

## Introduction

The ovarian response is a vital determinant of reproductive milestones, and ovarian function declines with age [1]. Along with economic development, the delay in childbearing has become a societal norm. As a result, more women are facing subfertility due to poor ovarian response (POR) and then seek medical help to conceive [2]. In vitro fertilization (IVF) is now the treatment of choice for women over 40 years old, and it is estimated that 17.9% of all IVF cycles are performed in these women [3]. In IVF cycles, the major factor for achieving pregnancy is ovarian responsiveness to controlled ovarian stimulation with gonadotropins. POR leads to insufficient retrieval of mature oocytes, high cycle cancellation and low pregnancy rates in IVF, thus becoming a challenge in assisted reproduction [4]. The occurrence of POR in IVF cycles ranges between 9% and 24% [5]. This wide range is caused by the lack of consensus regarding the definition of POR. The Bologna criteria were the first international consensus to define POR. In 2016, a new classification named POSEIDON was introduced, providing a more detailed stratification of POR patients. Compared with the Bologna criteria, the POSEIDON classification reduces the heterogeneity and promotes individualized treatment in these patients. At present, the management and treatment of patients with POR is still a debated issue in IVF. To overcome this challenge and achieve better clinical outcomes, many doctors have tried various controlled ovarian hyperstimulation protocols and strategies to improve pregnancy rates in patients with POR undergoing IVF procedures. However, the success rate remains low; the live birth rate per cycle is approximately 6% in this population [6]. According to the last Cochrane meta-analysis, there is insufficient evidence to support the routine use of any particular intervention in the management of poor responders [7], and there is a lack of clinical guidelines for the most optimal treatment of POR [8].

At present, the most prevalent approaches for treating POR patients are the routine short protocol and the mild stimulation protocol. Both have their own advantages and disadvantages. Neither of these protocols has been especially effective in improving IVF outcomes in these patients. To compensate for these shortcomings, we attempted to modify the routine short protocol to a flexible short protocol (FSP). This protocol may reduce the incidence of a premature luteinizing hormone (LH) surge and may reduce the cycle cancellation rate associated with the mild stimulation protocol [9]; furthermore, the time required for stimulation treatment and the dose of gonadotropins are significantly reduced in the FSP compared with the routine short protocol. This study explored the application value of the FSP in IVF and retrospectively analyzed the routine short protocol, mild stimulation protocol and FSP to find a new suitable ovarian stimulation protocol for POR patients over 40 years old.

## Materials and methods

### Study setting and population

All patients aged over 40 years who were referred to the IVF program of the Second Affiliated Hospital of Nanjing Medical University to undergo IVF cycles from January 2015 to June 2019 were eligible for this retrospective study, and the data were retrieved from patients’ medical records. The study was approved by the Reproductive Medicine Ethics Committee of the Second Affiliated Hospital of Nanjing Medical University (serial number: KY 01; date: November 2, 2014). All participants were informed of the three stimulation protocols after counseling for infertility treatments and IVF procedures. The patients were divided into one of the three groups according to the professional judgment of the experts and the wishes of the patients. A comparison was made between the three different stimulation protocols. The baseline characteristics of the three groups were similar. All women met the requirement of being over 40 years old; at the same time, they were categorized as poor responders according to the POSEIDON criteria [10] ( AFC < 5, AMH < 1.2 ng/mL). The exclusion criteria were as follows: (1) patients with systemic diseases who were unable to tolerate pregnancy and (2) patients with endometrial abnormalities such as uterine adhesions or endometrial polyps.

### Ovarian stimulation protocols

#### Mild stimulation protocol

**Fig. 1:**
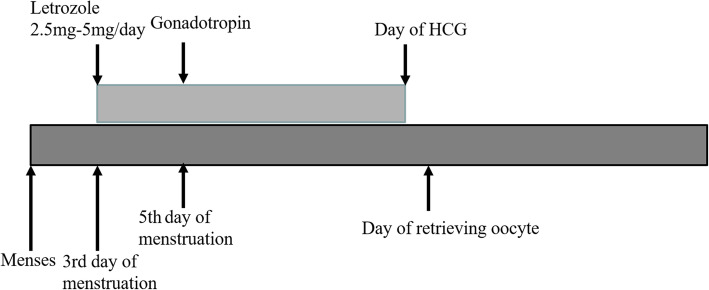
The mild stimulation protocol group

In women treated with the mild stimulation protocol, anti-estrogenic drugs, including letrozole, were administered orally from day three in the cycle until the day before the hCG trigger. Low doses of gonadotrophins, including human menopausal gonadotropin (HMG) or follicle-stimulating hormone (FSH), were administered in the form of injections starting on the 5th day of menstruation, and the dosage varied between 75 and 150 IU/day depending on follicular development. Monitoring of follicular development with ultrasound was initiated on day five of the menstrual cycle, and starting from this stage, the dose of gonadotrophins was adjusted depending on the individual response of each patient. When one or more follicles reached 18 mm in diameter, human chorionic gonadotropin hormone (hCG 10,000 IU) was administered intramuscularly to trigger oocyte maturation. Oocytes were retrieved 36 hours after the hCG trigger. The details could be seen in Fig. 1.

#### Routine short protocol

**Fig. 2:**
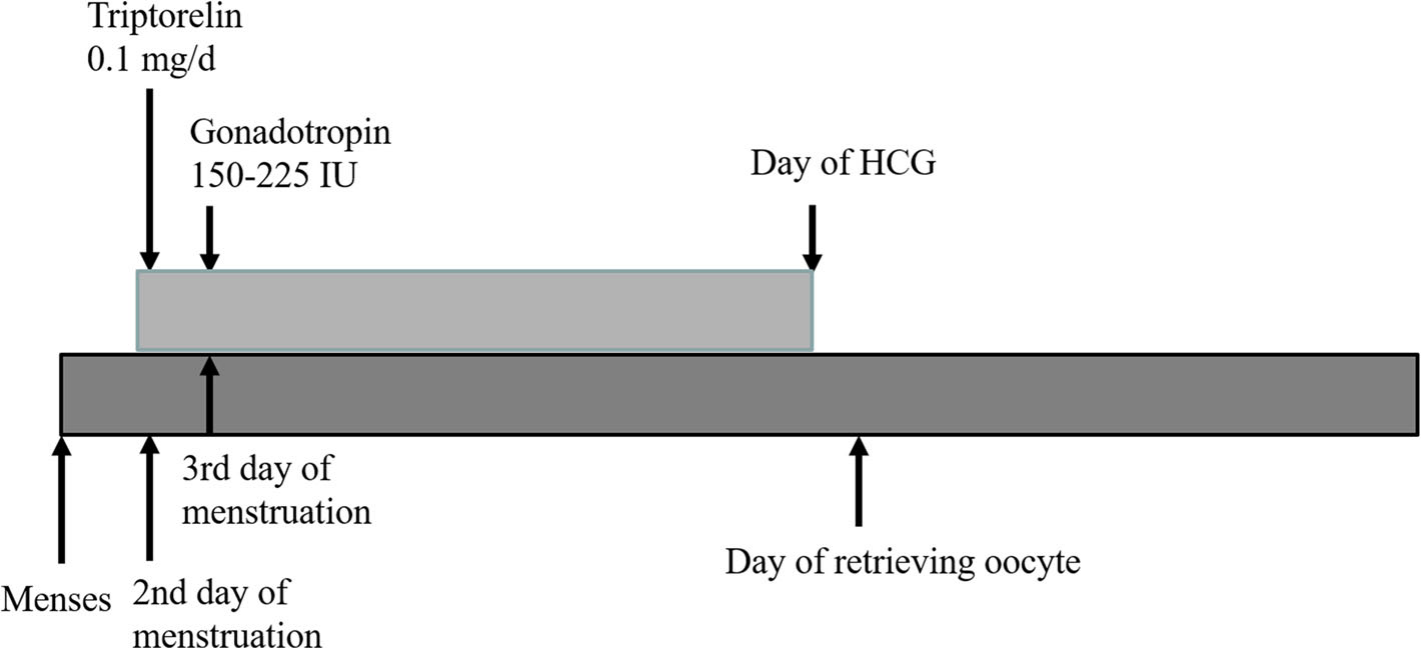
The routine short protocol group

Triptorelin (gonadotrophin-releasing hormon,0.1 mg/d) was injected subcutaneously from the second day of the menstruation cycle and continuing until the day of hCG administration, while gonadotropin was administered from day 3. The initial dosage of gonadotropin was 150–225 IU daily for the first 5 days, and then the dose was adjusted according to follicular development. The details could be seen in Fig. 2.

#### Flexible short protocol

**Fig. 3:**
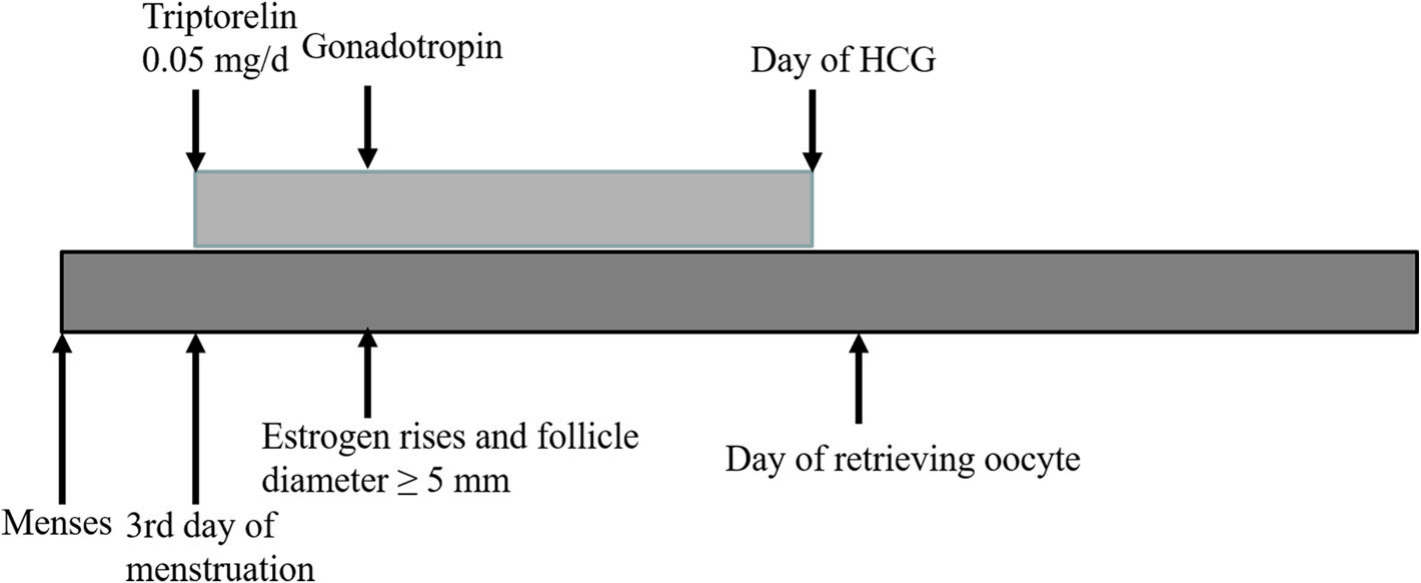
The flexible short protocol group

Triptorelin (0.05 mg/d) was injected from the third day of the menstruation cycle and continued until the day of hCG administration, while gonadotropin (FSH) was administered when estrogen began to rise and at least one follicle developed to 5 mm in diameter (follicular development was monitored by ultrasound every other day during this period). The gonadotropin injection starting time was more flexible, from the fifth to tenth day of menstruation to the day of hCG administration, and the minimal number of days of gonadotropin administration was four. The details could be seen in Fig. 3.

### Embryo culture

Cumulus-oocyte complexes (COCs) were collected by transvaginal aspiration 36 h after hCG administration. Oocytes were fertilized by either conventional IVF or ICSI according to the results of semen analysis. Embryo quality was evaluated daily according to The Istanbul consensus workshop on embryo assessment [11]. All the high-quality embryos were transferred or frozen by vitrification on the third day after oocyte retrieval. Usually, if there were more than six high-quality embryos, or if there were no high-quality embryos on day 3, culture extension to day 5 or day 6 was performed. Blastocysts were graded on the basis of the expansion of the blastocoel and the number and cohesiveness of the inner cell mass (ICM) and trophectoderm (TE) cells according to Gardner’s criteria [12].

### Embryo transfer

Ultrasound-guided fresh embryo transfer (ET) was performed on day 3 or day 5, with a maximum of 3 embryos transferred. However, if patients had serum progesterone levels > 2.0 ng/ml on the day of hCG administration or if they demonstrated risk of ovarian hyperstimulation syndrome, fresh embryo transfer was cancelled. For frozen embryo transfer cycles, according to the routine of the reproductive center or the patient’s individual preference, endometrial preparation was carried out with individualized schemes, such as the natural cycle and hormone replacement cycle.

### Outcome measures

The primary endpoints were the live birth rate (number of live birth cycles divided by the number of embryo transfer cycles), cumulative pregnancy rate (number of clinical pregnancies divided by the total number of patients) and clinical pregnancy rate (number of clinical pregnancies divided by the number of embryo transfer cycles), and the secondary outcomes were the total dose of gonadotropins used, the rate of high-quality embryos and the serum estradiol and luteinizing hormone levels on the last day of stimulation.

### Statistical analysis

Statistical analysis was performed with SPSS 18.0 (SPSS, USA). The data are shown as the mean ± SD and were analyzed by one-way analysis of variance. The chi-square or Fisher’s exact test was used to analyze the pregnancy outcomes. *P* < 0.05 was considered statistically significant.

## Results

Among the 488 cycles, there was no moderate/severe ovarian hyperstimulation, and 2 cycles demonstrated a premature LH surge in the mild stimulation protocol group. The baseline characteristics of patients in the three groups are shown in Table 1. The mean age of female patients was 44.43 ± 3.32 years in the FSP group, 43.99 ± 3.38 years in the routine short protocol group, and 44.74 ± 2.53 years in the mild protocol group. There was no significant difference in age, number of years of infertility, mean anti-Müllerian hormone level or basal AFC among the 3 groups. The baseline characteristics of the three groups were similar, that is, the data of the 3 groups were comparable.

**Table 1:** Baseline characteristics of the FSP, short protocol and mild protocol

Parameters	FSP (Flexible short protocol)	RSP (routine short protocol)	MSP (mild stimulation protocol)	P1	P2	P3
Number of cycles	237	76	175	NA	NA	NA
Age, years	44.43±3.32	43.99±3.38	44.74±2.53	0.274	0.317	0.198
Duration of infertility years	4.18±3.80	4.41±4.65	4.66±5.32	0.697	0.282	0.560
AMH(ng/ml)	0.53±0.57	0.46±0.53	0.50±0.57	0.472	0.595	0.713
Basal AFC	4.94±1.99	4.81±1.70	4.85±2.18	0.656	0.680	0.868

*FSP* flexible short protocol, *NA* not available

P1: comparisons between FLP and routine short protocol groups

P2: comparisons between FLP and mild stimulation groups

P3: comparisons between the three groups

Data are expressed as mean ± SD. *P*<0.05 significantly different from control group

As shown in Table 2 and 52 cycles ended with no oocyte retrievals (28 in the FSP group, 4 in the routine short stimulation group and 20 in the mild stimulation group). There were no significant differences in insemination modes among the three groups. The mean total amount of gonadotropins used was 1669.46 ± 981.15 IU in the FSP group, 2394.74 ± 997.27 IU in the routine short protocol group and 1836.20 ± 1070.27 IU in the mild stimulation protocol group (*P* < 0.001). There were significant differences in the duration of ovarian stimulation among the three groups. The length of stimulation in the FSP group was significantly shorter than that in the other two groups. At the same time, the E2 levels on the day of hCG administration were lower in the FSP group than in the RSP group. This was consistent with the result of the average number of oocytes retrieved. The LH levels on the day of hCG administration were 5.40 ± 9.50 in the FSP group, and these levels were significantly lower than those in the mild stimulation protocol group.

**Table 2:** Clinical outcomes of the FSP, short protocol and mild protocol

Parameters	FSP (Flexible short protocol)	RSP (routine short protocol)	MSP (mild stimulation protocol)	P1		P2		P3
Number of initiated cycles	237	76	175	NA		NA		NA
Number of cycles with oocytes retrievals	209	72	153	NA		NA		NA
Insemination modes								
IVF	89.95% (188/209)	91.67% (66/72)	90.20% (138/153)	0.670		0.939		0.912
ICSI	10.05% (21/209)	8.33% (6/72)	9.80% (15/153)	0.670		0.939		0.921
Mean total amount of gonadotropins (IU)	1669.46 ± 981.15	2394.74 ± 997.27	1836.20 ± 1070.27	0.000		0.101		0.000
Duration of ovarian stimulation (days)	7.47 ± 3.50	9.25 ± 4.21	9.48 ± 3.96	0.000		0.000		0.000
Serum E2 (pg/ml) on day of HCG	1094.12 ± 942.08	2120.51 ± 1310.94	922.39 ± 957.23	0.000		0.104		0.000
Serum LH (pg/ml) on day of HCG	5.40 ± 9.50	5.55 ± 3.00	11.04 ± 10.07	0.899		0.000		0.000
Serum P (pg/ml) on day of HCG	1.11 ± 1.60	1.35 ± 1.50	1.72 ± 2.73	0.383		0.005		0.019

*FSP* flexible short protocol, *NA* not available

P1: comparisons between FLP and routine short protocol groups

P2: comparisons between FLP and mild stimulation groups; P3: comparisons between the three groups

Data are expressed as mean ± SD. *P* < 0.05 significantly different from control group

The laboratory results are shown in Table 3. The average number of oocytes retrieved in the FSP and MSP groups was lower than that in the RSP group (2.19 and 2.13 vs. 3.53, respectively, *P* < = 0.001). The rate of 2PN was 76.48 ± 31.80 in the FSP group, 73.94 ± 31.44 in the routine short protocol group and 66.29 ± 38.62 in the mild stimulation protocol group. The rate of high-quality embryos in the FSP group was higher than that in the mild stimulation protocol group. There were 22 cycles with no transferable embryos in the FSP group, 4 in the routine short protocol group and 9 in the mild stimulation protocol group. There were 34 fresh embryo transfer cycles in the FSP group, 15 in the RSP group and 17 in the MSP group. A total of 255 embryos were transferred in the FSP group, 103 in the routine short protocol group and 181 in the mild stimulation protocol group. More importantly, the clinical pregnancy rate was 21.62% (32/148) in the FSP group, and the rate was significantly higher than that in the other two groups (*P* < 0.05), as was the cumulative pregnancy rate. There was no significant difference in the miscarriage rate among the three groups. A trend of a higher live birth rate (11.49%) was observed in the FSP group than in the other 2 groups (RSP: 8.77%; MSP: 4.72%), but the difference was not statistically significant. There were 2 patients with twin pregnancies in the FSP group, 1 in the routine short protocol group and 1 in the mild stimulation protocol group. The implantation rate was 13.33% in the FSP group, 8.74% in the routine short protocol group and 5.52% in the mild stimulation protocol group. The FSP group had a significantly higher implantation rate than the MSP group (*P* = 0.024).

**Table 3:** Laboratory outcomes of the FSP, short protocol and mild protocol

Parameters		FSP (Flexible short protocol)	RSP (routine short protocol)	MSP (mild stimulation protocol)		P1		P2		P3
Number of retrieved oocytes		2.19 ± 1.95	3.53 ± 2.00	2.13 ± 1.81	0.000			0.743		0.000
2PN rate		76.48 ± 31.80	73.94 ± 31.44	66.29 ± 38.62	0.590			0.006		0.021
Number of high- quality embryos		1.25 ± 1.23	1.58 ± 1.30	0.99 ± 1.22	0.054			0.061		0.004
Rate of high-quality embryo (%)		62.84 ± 42.48	56.12 ± 41.90	50.71 ± 44.05	0.283			0.011		0.039
Cycles with transferable embryos		187	68	144	NA			NA		NA
Embryo transfer cycles		148	57	106	NA			NA		NA
Fresh embryo transfer cycles		22.97% (34/148)	26.32% (15/57)	16.04% (17/106)	0.615			0.174		0.239
Frozen embryo transfer cycles		77.03% (114/148)	73.68% (42/57)	83.96% (89/106)	0.615			0.174		0.239
Mean of embryos Transferred		1.72	1.81	1.71	NA			NA		NA
Clinical pregnancy rate (%)		21.62 (32/148)	14.04 (8/57)	8.49 (9/106)	0.219			0.005		0.017
Cumulative pregnancy rate (%)		25.81 (32/124)	17.78 (8/45)	9.28 (9/97)	0.278			0.002		0.007
Implantation rate (%)		13.33 (34/255)	8.74 (9/103)	5.52 (10/181)	0.226			0.008		0.024
Miscarriage rate (%)		46.88 (15/32)	37.50 (3/8)	44.44 (4/9)	0.634			0.897		0.892
Live birth rate (%)		11.49 (17/148)	8.77 (5/57)	4.72 (5/106)	0.574			0.059		0.168

*FSP* flexible short protocol, *NA* not available

P1: comparisons between FLP and routine short protocol groups

P2: comparisons between FLP and mild stimulation groups

P3: comparisons between the three groups

Clinical pregnancy rate: number of clinical pregnancies divided by the number of embryo transfer cycles. Cumulative pregnancy rate: number of clinical pregnancies divided by the number of all patients. Implantation rate: number of gestational sacs divided by the number of embryos that were transferred. Miscarriage rate: number of miscarriage cycles divided by the number of clinical pregnancies. Live birth rate: number of live birth cycles divided by the number of embryo transfer cycles. Data are expressed as mean ± SD. *P* < 0.05 significantly different from control group

## Discussion

### Main results

The present retrospective analysis shows that the FSP may be a useful stimulation protocol in women with poor ovarian response over 40 years old. Compared with the routine short protocol, the FSP delayed the start-up time of gonadotropin administration and reduced gonadotropin usage. At the same time, compared with the mild stimulation protocol, the FSP improved the quality of oocytes and reduced the probability of premature ovulation, as this approach inhibited the premature LH surge to a certain extent. Furthermore, the FSP had a higher 2PN rate and a higher rate of high-quality embryos, that is, the FSP improved the quality of oocytes. Additionally, the FSP had a significantly higher pregnancy rate and implantation rate. Also, it had a trend of a higher live birth rate than the other two groups, but the difference was not statistically significant, which may be because there were too few numbers of live births.

### Interpretation of the results

The classical short protocol has been used in the clinic for 20 years and uses the flare-up effect of gonadotropin releasing hormone agonist (GnRH-a) on the pituitary at the initial stage to induce the rapid release of gonadotropins in the pituitary, thereby enhancing the recruitment of early follicles. This protocol is especially recommended for patients with POR [13]. However, the RSP seems to be less effective based on the number of oocytes retrieved compared with the long GnRH-agonist protocol [14]. Documents have shown that the cumulative pregnancy rate of the mild stimulation protocol is similar to that of the conventional ovarian stimulation protocol, but the time required for treatment and the dose of gonadotropins are significantly reduced, which reduces the economic burden of patients [15]. The disadvantages of the mild stimulation protocol are the lower number of oocytes and higher cancellation rate, as this approach does not inhibit the premature LH surge. In the FSP and RSP, the LH level initially increases sharply, then plateaus and then becomes suppressed days after the administration of GnRH agonists [16]. Therefore, the FSP makes up for the disadvantages of the MSP. At the same time, although the short protocol had the disadvantage of fewer oocytes retrieved, the FSP could obtain higher quality oocytes compared with the RSP, and we achieved excellent success rates with this strategy in women with POR.

As each menstrual cycle begins, the intercyclic increase in FSH recruits intermediately mature (2–5 mm diameter) follicles to enter the initial stages of preovulatory development [17]. In the FSP, the use of GnRH-a can stimulate the release of endogenous FSH and allow endogenous intercyclic increase in FSH to be utilized for follicle stimulation. At the same time, GnRH-a may suppress the LH flare in the mid-late follicular phase due to its downregulating effect on the pituitary gland [18]. According to previous studies, premature luteinization has been observed to occur more frequently in older or POR patients [19]. Defects in ovarian responsiveness to FSH include reduced gonadotrophin surge-attenuating factor (GnSAF) production, which contributes to the inability to control the secretion of LH [20]. Administration of GnRH-a leads to a reversible blockade of pituitary function after an initial stimulatory phase. GnRH-a may suppress GnRH receptors and cause inhibition of post receptor events [18]. Thus, GnRH-a contributes to the reduction of bioactive LH levels in serum and allows multiple follicular development, avoiding the risk of an LH surge and thereby avoiding premature ovulation [18]. In this study, compared with the mild stimulation protocol, the FSP may reduce the cycle cancellation rate by suppressing the LH surge.

According to a previous study, cyclic follicle recruitment and initial stages of dominant follicle selection can proceed within the natural cycle, and the use of exogenous FSH for inducing multiple follicle development can be restricted during the mid-late follicular phase [21]. Therefore, gonadotropin (FSH) was administered when the estrogen level increased, and at least one follicle developed to 5 mm in diameter. The increase in estrogen indicates that these follicles begin to develop under the stimulation of endogenous FSH. However, endogenous FSH may lead to early follicular selection and asynchronous growth. Marked follicular size discrepancies lead to decreased oocyte maturation and fertilization potential. To accomplish simultaneous maturation, follicles are required to grow in coordination with exogenous gonadotropins [22]. Exogenous gonadotropin (FSH) can further promote the development of high-quality basic follicles under natural selection and increase synchronous follicle growth, which is a “delayed start” protocol with Gn. Indeed, patients with POR may not respond to stimulation and do not benefit from taking high doses of exogenous gonadotrophins during stimulated IVF [23]. The FSP reduces the dose of Gn, which is a less costly procedure. However, there were more cycles with no oocytes retrieved and no transferable embryos in the FSP group compared with the other two groups, it appears that for this cohort, there may be a potential selective effect where a subset of patients that produce no oocytes, or, embryos, do not contribute to pregnancy outcomes. Thus, the FSP protocol may be “weeding out” some patients and thus gain an artificial boost in outcomes. Meanwhile, the number of cycles with no oocyte retrievals and no transferable embryos was small in the RSP (four), the proportion was low, and the representativeness was insufficient. This is challenging to determine directly, the effect may be selective due to removing patients that do not have oocytes retrieved and separately, do not have embryos to transfer, but it is impossible to be ignored. In the future, a larger sample of prospective randomized controlled trials was needed to obtain more accurate results.

### Strengths and limitations

The advantage of this study lies in its larger sample size compared with other similar studies, and it is a successful modification of the routine short protocol. This new protocol enables more flexibility and is of emerging interest in daily practice. This study also has limitations. First, this was a retrospective study, the research time was short, and the types of stimulation protocols were limited. An ideal protocol for poor responders has not been determined. Therefore, the comparison of various ovarian stimulation protocols should be further investigated in well-designed, prospective randomized controlled clinical trials in the future. Second, this work was conducted in a specific subgroup of patients. Further research is required to determine whether the FSP can be successfully applied to younger POR patients.

## Conclusions

The FSP may be a good choice for patients with POR of advanced age (> 40 years old), as it allows patients to use a lower dose of Gn and achieve a higher pregnancy rate and implantation rate. However, further prospective clinical research is needed to provide more substantial evidence.

## Data Availability

The datasets used and/or analyzed during the current study are available from the corresponding author on reasonable request.

## Abbreviations

ART: Assisted Reproductive Technology
POR: Poor Ovarian Response
FSP: Flexible Short Protocol
LH: Luteinizing Hormone
IVF: In Vitro Fertilization
HMG: Human Menopausal Gonadotropin
FSH: Follicle-Stimulating Hormone
hCG: Human Chorionic Gonadotropin
COC: Cumulus-Oocyte Complexes
ICM: Inner Cell Mass
TE: Trophectoderm
ET: Embryo Transfer
GnRH-a: Gonadotropin Releasing Hormone agonist
GnSAF: Gonadotrophin Surge-Attenuating Factor
